# Rapid Access Macular Screening and Evaluation: A Datasheet for a Dataset to Diagnose and Triage Macular Diseases

**DOI:** 10.1016/j.xops.2026.101253

**Published:** 2026-05-27

**Authors:** Ariel Yuhan Ong, Reena Chopra, Henry David Jeffry Hogg, Alastair K. Denniston, Pearse A. Keane

**Affiliations:** 1 Moorfields Eye Hospital NHS Foundation Trust, London, United Kingdom; 2 Institute of Ophthalmology, University College London, London, United Kingdom; 3 NIHR Moorfields Biomedical Research Centre, London, United Kingdom; 4 Oxford Eye Hospital, Oxford University Hospitals NHS Foundation Trust, Oxford, United Kingdom; 5 Topcon Healthcare Inc, San Diego, California; 6 College of Medicine and Health, University of Birmingham, Birmingham, United Kingdom; 7 NIHR Birmingham Biomedical Research Centre, Birmingham, United Kingdom; 8 University Hospitals Birmingham NHS Foundation Trust, Birmingham, United Kingdom

**Keywords:** Dataset, Macula, OCT, Retinal imaging

## Abstract

**Purpose:**

Macular disease can cause significant visual morbidity. Timely and accurate diagnosis and management is paramount. However, there is a lack of high-quality multimodal datasets reflective of real-world clinical practice to support research in this area. We present a large longitudinal real-world dataset that aims to address this gap.

**Design:**

Datasheet describing a multimodal dataset of routinely collected ophthalmic data focusing on possible macular disease.

**Participants:**

Adult patients (aged ≥18 years) attending the retina service at Moorfields Eye Hospital National Health Service Foundation Trust for the first time from February 1, 2017 to July 31, 2025 with macula-centered OCT scans (Topcon or Heidelberg).

**Methods:**

Data were curated from the INSIGHT Health Data Research Hub, one of the world’s largest ophthalmic imaging bioresources, which aims to provide researchers with controlled access to anonymized routinely collected data. Clinical and imaging data derived from routine clinical care were exported, processed, and deidentified for secondary research use.

**Main Outcome Measures:**

This datasheet describes the demographic, clinical, and imaging metadata of the dataset, including a transparent overview of its strengths and weaknesses.

**Results:**

Rapid Access Macular Screening and Evaluation (RAMSEs) is a large and diverse real-world dataset. It was specifically designed to facilitate the diagnosis and triage of possible macular disease by providing access to multimodal imaging and clinical metadata. The current version of this dataset (time-locked as of July 2025) consists of retinal images and linked sociodemographic and clinical metadata from 85 444 patients with a median age of 63 (interquartile range 50–75) and a fairly even gender distribution (52.2% female). It comprises >4.9 million multimodal ophthalmic images (e.g. color fundus photographs, ultra-widefield imaging, fundus autofluorescence), including >1.4 million macula-centered OCT scans.

**Conclusions:**

We have developed a large multimodal real-world dataset, which was designed to address existing gaps in dataset size, disease distribution, and key clinical and sociodemographic metadata for possible macular disease. This valuable longitudinal resource can serve multiple purposes, including the development or robust clinical validation of artificial intelligence models, facilitating insights into real-world patient pathways and outcomes, or enabling research relating to epidemiology or big data analytics. This dataset may be made available through INSIGHT via a structured application process.

**Financial Disclosure(s):**

Proprietary or commercial disclosure may be found in the Footnotes and Disclosures at the end of this article.

Macular disease encompasses a range of conditions affecting the central retina, which is essential for high-resolution visual tasks such as reading, recognizing faces, and driving. In the United Kingdom (UK) alone, 1.5 million individuals (or approximately 2.2% of the population) are affected by macular disease,^1^ and the burden of disease is projected to rise as the population continues to age. Examples include age-related macular degeneration (AMD), one of the leading causes of visual impairment and blindness globally;^2^ macular edema, which can arise from diabetes-related retinal microvascular changes, postoperative inflammation, or retinal vascular occlusion; and macular hole and epiretinal membrane secondary to vitreoretinal interface disorders.

Regardless of the specific condition, timely and accurate diagnosis and management are paramount. However, despite rapid advancements in diagnostic imaging technologies, challenges in delivering timely and equitable care remain. Ophthalmology has become the busiest outpatient specialty in the National Health Service (NHS) today, with close to 10 million appointments in England alone annually as of 2024-5.^3^ Service demand is projected to continue rising by 30%–40% over the coming decade,^4^ driven by demographic trends and the increasing prevalence of chronic diseases such as diabetes. This capacity-demand imbalance will exacerbate the growing risk of irreversible sight loss from treatment delays,^5^, ^6^, ^7^ affecting quality of life for patients and carers^8^, ^9^, ^10^ and posing a significant economic burden to individuals, health care services, and society.^11^^,^^12^

Artificial intelligence (AI) has emerged as a promising solution to address these challenges. Artificial intelligence has significant potential in terms of enhancing diagnostic accuracy, optimizing treatment planning or delivery, streamlining clinical workflows, and improving access to care.^13^ However, for AI models to be clinically useful, robust datasets that represent real-world populations and conditions are required to train these models and/or support clinical validation. This will help ensure that they meet stringent safety, performance, and health equity criteria before real-world deployment. To this end, we have developed Rapid Access Macular Screening and Evaluation (RAMSEs), a large and diverse dataset specifically designed to facilitate the diagnosis and triage of macular diseases.

In this paper, we will discuss the rationale for dataset creation, strengths, use cases, ethical considerations, data processing, and dataset composition (including relevant participant metadata), with the aim of establishing RAMSEs as a valuable real-world resource for AI research and development for macular disease. Having considered various approaches to dataset documentation, we have adopted a hybrid strategy that integrates recommendations from the STANDING Together initiative^14^ and the “Datasheets for Datasets”^15^ framework to facilitate transparent and comprehensive reporting.

## Motivation for Dataset Creation

At present, there are a limited number of open or partially open-access ophthalmic imaging datasets for macular diseases.^16^^,^^17^ These datasets are further limited by their small cohort sizes (typically counted on a B-scan or image level, rather than an eye or patient level) and limited representation of disease classes. Most datasets restrict inclusion to a few select disease categories, with conditions such as AMD and diabetic eye disease being overrepresented in relation to others. Such highly curated datasets often do not necessarily reflect the spectrum of macular diseases, disease severity, and image quality present in real-world clinical practice, which risks introducing spectrum bias.^16^^,^^18^ Inconsistent standards for image acquisition, image provision (in terms of format and resolution), and annotation may also affect the reproducibility and reliability of models trained on these datasets.^17^

At the same time, many datasets do not document critical metadata such as laterality, basic sociodemographic characteristics (age, sex, ethnicity)—both at the individual and aggregate level—or may lack diversity in their patient population.^16^^,^^19^ According to the MINimum Information (MINIMAR) for Medical AI Reporting standards, demographic variables including age, sex, race, ethnicity, and socioeconomic status should be reported at a minimum,^20^ to facilitate more granular understanding of AI models’ performance in diverse patient populations. Beyond evaluating overall model performance across the entire cohort, international consensus-based recommendations from the STANDING Together collaboration^14^ further emphasize the need to evaluate AI performance across these patient subgroups, to highlight and/or mitigate bias and help assess whether the AI is “safe on average, or safe for all.”^16^ This requires a sufficiently large and diverse dataset to enable adequately powered analyses.

## Purpose and Potential Use Cases

The RAMSEs dataset was created to bridge these gaps and address the pressing need for efficient and accurate triage and diagnosis pathways for macular disease. The primary purpose was to perform robust clinical validation of an OCT-based AI model that diagnoses and triages a range of macular diseases in a representative cohort of real-world patients (hereinafter referred to as the “Moorfields-Deepmind algorithm”).^21^ Importantly, the dataset enables but does not predefine diagnostic or triage modeling frameworks. These are derived modeling tasks based on imaging and clinical data rather than prespecified triage labels or simulation outputs.

Beyond this, the rich tabular and multimodal imaging data within RAMSEs can be used for developing and/or validating AI models from single imaging modalities or multimodal imaging; for conducting service evaluations to obtain insights into real-world patient pathways and treatment outcomes; or to facilitate research relating to epidemiology or big data analytics. Examples include analyses of longitudinal disease trajectories (e.g. imaging-defined progression of macular pathology), characterization of health care resource utilization patterns across clinical services, and real-world treatment outcome studies following intravitreal therapy. In addition, the dataset supports risk stratification and phenotyping approaches, including clustering of multimodal imaging features to identify clinically meaningful subgroups of macular disease.

## Data Collection

RAMSEs comprises a retrospective cohort of adult patients aged ≥18 years who have attended Moorfields Eye Hospital NHS Foundation Trust (MEH). MEH encompasses 27 networked centers serving a socioeconomically and ethnically diverse catchment of 6 million people across London, approximately 9% of the UK population. This includes a tertiary eye center, several district general hospitals, and multiple community-based diagnostic hubs.

To fulfill the purpose of facilitating the diagnosis and triage of people referred with possible macular conditions, participants were included if their first visit to the medical retina or vitreoretinal clinics fell between February 1, 2017 and July 31, 2025 (inclusive), and they had a macula-centered Topcon or Heidelberg OCT scan at this visit. These OCT device manufacturers were selected because the Moorfields-Deepmind algorithm was primarily trained on Topcon OCT scans and retrained on a small number of Heidelberg scans.^21^ In addition, these are the 2 most commonly used OCT devices at MEH. The current version of RAMSEs is a time-locked dataset, with data collection censored at July 31, 2025.

Eligible participants were identified from the INSIGHT Database (Moorfields) by filtering relevant service codes and clinic codes, and cross-referencing imaging records against the clinic visit date. INSIGHT is a health data research hub established under the auspices of Health Data Research UK and facilitates controlled access to anonymized clinical data (featuring both tabular and ophthalmic imaging data) obtained longitudinally from routine patient visits to MEH.^22^ In brief, the INSIGHT Database ingests patient-level data fields from the Moorfields Data Warehouse, including sociodemographic data from the patient administration system; ophthalmic clinical data from electronic health record (EHR) systems and specialized databases; and ophthalmic images and their associated metadata.

All data within the INSIGHT Database (and by extension RAMSEs) were collected as part of routine clinical care from patient visits to MEH and subsequently made available for approved secondary purposes such as research to improve patient care, in accordance with the NHS constitution. Patients may choose not to participate through the “National Data Opt-Out” service.^23^ As such, as of July 2025, INSIGHT covers approximately 93.0% of the catchment population (7.0% opt-out rate in London).^24^

## Data Preprocessing and Cleaning

Images were exported via automated pipelines that ingest and convert raw image files (e.g. color fundus photographs, OCT scans) from proprietary manufacturer-specific formats to Digital Imaging and Communications in Medicine (DICOM) standards. Deidentified images were subsequently linked to their corresponding clinical records. These processes use proprietary custom-built software tools created by Softwire Technology Limited for INSIGHT.

Tabular data extracted from 2 EHRs (Medisoft, Medisoft Ltd and OpenEyes, Apperta Foundation) and the Moorfields Data Warehouse were standardized to the INSIGHT data dictionary. Relevant metadata such as clinical and demographic attributes were mapped to common data terminologies where appropriate—for example, ethnicity codes were mapped to the categories defined in the NHS Data Dictionary,^25^ and procedures to Systematized Nomenclature of Medicine Clinical Terms (SNOMED-CT) codes.^26^

Data quality assurance was performed via automated rules-based processes. For each data field, customized validation rules defining permissible value sets, data types, and contextual thresholds are prespecified. This governs the systematic evaluation of each data field against the 7 Data Management Association dimensions (accuracy, completeness, consistency, validity, timeliness, uniqueness, integrity). Outliers were flagged for cleaning, with discrepancies resolved prior to dataset finalization.

For the purposes of this datasheet, data processing and analysis were conducted in Python (v3.12.4, Python Software Foundation). Sankey diagrams were generated using the *Plotly* library, while other figures were created using *Matplotlib*.

## Dataset Composition

### Composition of Groups and Relevant Attributes

RAMSEs comprises data from 85 444 adult patients who were seen for the first time in the MEH Medical Retina and Vitreoretinal services between February 1, 2017 and July 31, 2025.

The dataset includes the following patient attributes: age, sex, ethnicity, and socioeconomic status. These attributes may be associated with disparities in access to health care as well as risk and severity of disease. Age and sex were extracted directly from EHRs, reflecting prespecified administrative fields. Ethnicity was optionally self-reported by the individual at registration and classified according to the NHS Data Dictionary.^25^ Socioeconomic status was represented by the 2019 Index of Multiple Deprivation (IMD), which is the official small-area measure of relative deprivation in England and is measured based on various factors such as income, employment, and health. This was calculated by mapping patient postcodes to Lower-layer Super Output Areas via the Office of National Statistics Postcode Directory.^27^

**Figure 1:**
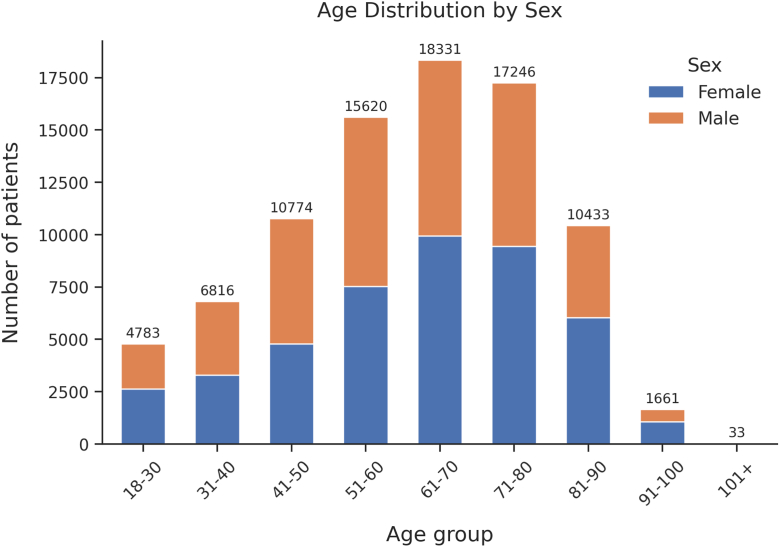
Age and sex distribution of all patients included in the RAMSEs cohort. RAMSEs = Rapid Access Macular Screening and Evaluation.

The median patient age at the baseline visit was 63 years (interquartile range [IQR] 50–75), with slightly more females than males (44 584, 52.2% female). The median IMD decile was 5 (IQR 3–7), with a right skew placing more patients in the more deprived deciles. Of patients with available ethnicity data, White was the commonest reported ethnic group (17 842, 20.9%), followed by Other Ethnic Groups (11 939, 14.0%). Sociodemographic data are presented in Table 1 and Figure 1.

**Table 1:** Demographic Characteristics of Patients in the RAMSEs Cohort

Category	Subcategory	N	Percentage (%)
Age (years)	18–30	4759	5.6
	31–40	6790	8.0
	41–50	10 738	12.6
	51–60	15 585	18.2
	61–70	18 293	21.4
	71–80	17 208	20.1
	81–90	10 388	12.2
	91–100	1650	1.9
	>100	33	0.0
Sex	Male	40 584	47.8
	Female	44 584	52.2
	Unknown	6	0.0
Ethnicity groups∗	White	17 842	20.9
	Asian or Asian British	9623	11.3
	Black or Black British	4906	5.7
	Other Ethnic Groups	11 939	14.0
	Mixed	591	0.7
	Unknown	40 657	47.4
Ethnicity subgroups∗	White - British	14 004	16.4
	White - Irish	721	0.8
	Asian or Asian British - Indian	4819	5.6
	Black or Black British - African	2394	2.8
	Black or Black British - Caribbean	1833	2.1
	Asian or Asian British - Pakistani	1240	1.5
	Asian or Asian British - Bangladeshi	1112	1.3
	White - any other White	3117	3.6
	Asian or Asian British - any other Asian	2452	2.9
	Other - Chinese	478	0.6
	Other - any other ethnic background	11 461	13.4
	Black or Black British - any other Black background	679	0.8
	Mixed - any other mixed background	216	0.3
	Mixed - White and Black Caribbean	165	0.2
	Mixed - White and Black African	96	0.1
	Mixed - White and Asian	114	0.1
	Unknown	40 543	47.4
Index of Multiple Deprivation (IMD)—presented as deciles to preserve anonymity	1 (most deprived)	1904	2.2
	2	10 619	12.4
	3	12 802	15.0
	4	11 126	13.0
	5	9327	10.9
	6	9305	10.9
	7	7656	9.0
	8	6466	7.5
	9	6285	7.4
	10 (least deprived)	5002	5.9
	Unknown	4972	5.8

RAMSEs = Rapid Access Macular Screening and Evaluation.

∗ Ethnicity groups and subgroups are presented as defined in the National Health Service Data Dictionary.

### Data Fields of Interest

Beyond the sociodemographic variables detailed above, the RAMSEs dataset captures multiple other clinically relevant data fields that characterize patients’ journeys across the care pathway, spanning referral, diagnosis, treatment, and outcomes. These include:

• Referral data
• Diagnosis
• Visual acuity (VA) data
• Multimodal ophthalmic imaging (currently OCT, color fundus photographs, pseudocolor imaging, autofluorescence, and fluorescein and indocyanine green angiography) and their associated imaging metadata
• Procedures including intravitreal injections and operations

#### Referral

**Figure 2:**
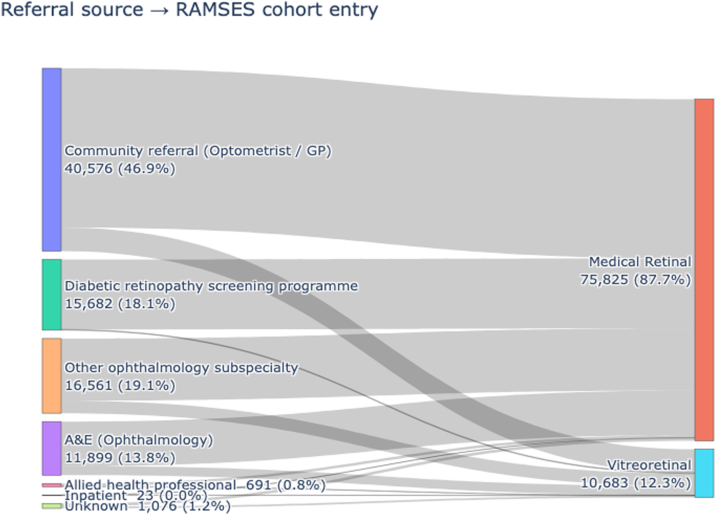
Sankey diagram illustrating the referral flow leading to the baseline appointment for patients in the RAMSEs cohort. Link widths are proportional to the number of patients. A&E = accident and emergency; RAMSEs = Rapid Access Macular Screening and Evaluation.

All included patients had a documented referral source, of which community referral from primary care was the most common (40 346, 47.2%), followed by referral from other ophthalmology subspecialties within MEH (e.g. glaucoma or general ophthalmology) (15 813, 18.5%), and referral from the national diabetic retinopathy (DR) screening program (15 650, 18.3%). The flow of referrals to the baseline Medical Retina or Vitreoretinal clinic appointment at cohort entry is shown in Figure 2.

#### VA

All VA measurements in the INSIGHT database were extracted as recorded in structured EHR data fields using multiple correction methods (glasses, contact lens, pinhole, formal refraction, autorefraction, and unaided) and multiple measurement methods, including logarithm of the minimum angle of resolution (logMAR)–based charts (e.g. ETDRS, crowded logMAR, COMPlog), Snellen meters, and letter-based formats. These readings were converted to logMAR units within INSIGHT for comparability. Non-numeric VA entries (counting fingers, hand movements, perception of light, and no perception of light) were converted to logMAR values of 2.1, 2.4, 2.7, and 3.0, respectively, for analysis, in accordance with conversions used in national database studies conducted by the Royal College of Ophthalmologists in the UK.^28^

Best documented VA at each event was summarized for this analysis. Of the 37 395 patients (74 694 eyes) with a VA reading at presentation, median best documented VA was 0.18 logMAR (IQR 0.00–0.30) across both eyes. Of these, 56 162 (75.2%) of eyes had good vision (defined as logMAR ≤0.30) and 6685 (8.9%) had poor vision (defined as logMAR ≥1.00). The median presenting VA in the right (0.18, IQR 0.00–0.30) and left eyes (0.18, IQR 0.00–0.40) was similar. In total, there were 1 062 777 VA records across all eyes over the 8.5-year study period.

#### Ophthalmic Imaging Modalities

**Figure 3:**
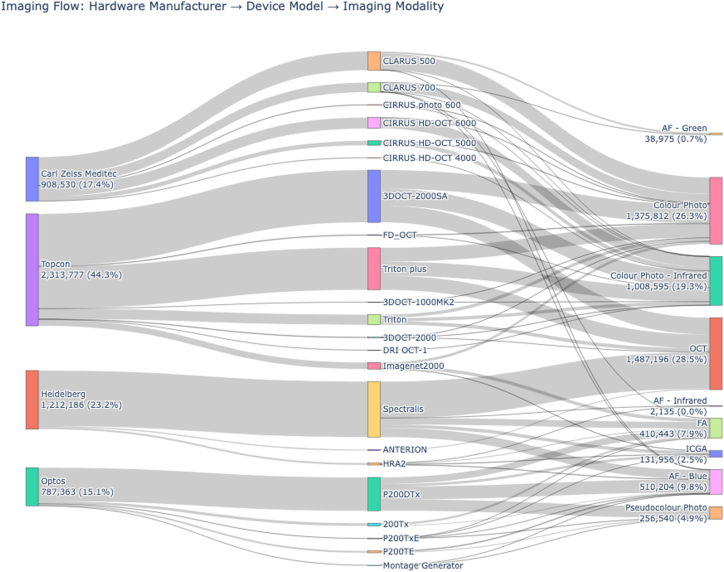
Sankey diagram illustrating the flow of images from imaging hardware manufacturers to device model to imaging modality. Link widths are proportional to the number of images acquired, highlighting the relative contribution of different manufacturers and device models to each modality within the dataset. AF = autofluorescence; FA = fluorescein angiography; ICGA = indocyanine green angiography.

Overall, 4.9 million ophthalmic images were included in the dataset. This included just >1.42 million OCT scans, with macula-centered OCT scans comprising the majority (1.3 million). Other retinal imaging modalities included color fundus photographs, infrared images, autofluorescence, ultra-widefield pseudocolor imaging, fluorescein angiography, and indocyanine green angiography. A small number of anterior segment scans were also included. Data on imaging modalities are presented in Table 2. Figure 3 shows the distribution of imaging modalities by hardware manufacturer and device model.

**Table 2:** Total Counts for Each Imaging Modality by Number of Eyes, Number of Patients, and Number of Images over the RAMSEs Study Period

Imaging Modality	Number of Eyes	Number of Patients	Total Number of Images
Macula OCT	169 346	85 444	1 336 805
Disc OCT	31 795	16 843	86 788
Anterior segment OCT	9321	7115	43 767
Color photographs	135 943	67 588	1 368 891
Infrared image	131 665	66 329	1 004 658
Pseudocolor image	73 301	36 874	252 239
Autofluorescence - blue	108 960	55 495	500 160
Autofluorescence - green	17 729	9176	38 872
Autofluorescence - infrared	362	226	2086
Fluorescein angiography	22 195	11 245	Images: 404 144 Series: 28 068
Indocyanine green angiography	6919	3500	Images: 128 357 Series: 8356

RAMSEs = Rapid Access Macular Screening and Evaluation.

**Figure 4:**
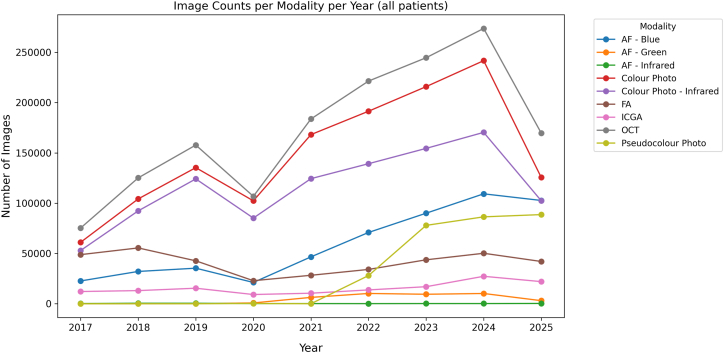
Image counts per retinal imaging modality from 2017–2025. Caveat: data from 2025 are under a full year (cutoff date on July 31, 2025). AF = autofluorescence; FA = fluorescein angiography; ICGA = indocyanine green angiography.

Figure 4 shows temporal trends in image acquisition stratified by imaging modality. In general, image counts increased from 2017–2019 across most imaging modalities, fell sharply during the coronavirus disease 2019 pandemic in 2020, and rose progressively from 2021 onward. The only exceptions were indocyanine green angiography (which remained fairly stable over time), and fluorescein angiography, which showed a sustained decline from 2017–2020 and only a slight subsequent increase thereafter.

All patients had macular OCT scans (paired with near infrared images) at their baseline visit. Overall, each eye had a median of 4 OCT volumes (IQR 2–9) throughout the study period. Five OCT devices were used, with Spectralis (Heidelberg Engineering GmbH) being most common at the baseline visit (78 880, 46.7%) and over the entire study period (684 409, 51.2%), followed by 3DOCT-2000SA (Topcon Corporation) (Table 3). There was an increasing trend in the use of Spectralis, Triton, and Triton Plus OCT scans over the study period, while the use of 3DOCT-2000SA declined overall.

**Table 3:** Counts for Macula-Centered OCT Scans Categorized by Device Model at Baseline Visit and Overall

Hardware Manufacturer	Hardware Model	Image Counts at Baseline Visit (%)	Total Image Counts Overall (%)
Topcon	3DOCT-2000SA	56 361 (33.3)	314 867 (23.6)
	Triton plus	23 081 (13.7)	267 916 (20.0)
	Triton	9971 (5.9)	66 340 (5.0)
	3DOCT-2000	745 (0.4)	3273 (0.2)
Heidelberg	Spectralis	78 880 (46.7)	684 409 (51.2)

#### Diagnosis Labels

RAMSEs includes eyes with a spectrum of macular pathology as well as those with normal imaging and nonmacular diagnoses. This reflects real-world clinical practice, in which patients may present with incidental findings, no detectable abnormalities, or unilateral disease with unaffected fellow eyes. Only diagnosis labels obtained from structured data fields are available for this iteration of RAMSEs; further work to supplement missing labels via data extraction from unstructured records is ongoing.^29^

Structured macular disease labels were available for 21 547 patients (25.2% of the cohort) at the baseline visit. The most common diagnosis at presentation was macular edema (17 489 eyes of 12 445 patients), which comprised diabetic macular edema, retinal vein occlusion-associated macular edema, uveitic macular edema, postoperative macular edema, and others. This was followed by macular neovascularization (4764 eyes of 4174 patients) which was predominantly due to neovascular AMD, but also included inflammatory and idiopathic causes. Other common diagnoses include: epiretinal membrane (1906 eyes of 1643 patients), dry AMD (1229 eyes of 760 patients), full-thickness macular hole (1025 eyes of 983 patients), and central serous retinopathy (881 eyes of 709 patients). Less common conditions such as myopic macular degeneration (132 eyes of 86 patients), retinal artery occlusion (88 eyes of 84 patients), macular telangiectasia (76 eyes of 45 patients), and hydroxychloroquine retinopathy (2 eyes of 1 patient) were present as well.

In total, 22.7% (19 393) of the cohort were reviewed for DR screening, of which 12 865 eyes of 8801 patients (45.4% of screening cohort) had diabetic macular edema. This was accompanied by background DR (16 677 eyes of 9573 patients), preproliferative DR (10 499 eyes of 6087 patients), and proliferative DR (3232 eyes of 2124 patients); the remainder had no DR.

#### Intravitreal Injections

Within this dataset, 13 650 eyes of 11 124 patients were treated with a total of 149 298 intravitreal injections. Two thousand five hundred twenty-six patients (22.7%) received bilateral injections. Over the study period, a median of 7 (IQR 4–14) injections were delivered per eye, and a median of 8 (IQR 4–17) injections were delivered per patient overall. The most common indications for treatment at any time point throughout the study period were neovascular AMD (5458 eyes, 37.8%), followed by diabetic macular edema (2908 eyes, 19.9%), macular edema secondary to branch retinal vein occlusion (1796, 12.3%) or central retinal vein occlusion (1080 eyes, 7.4%), uveitis-related macular edema (566 eyes, 3.9%), myopic macular neovascularization (428, 2.9%), and others.

#### Follow-Up

The median duration of follow-up was 1.6 years (IQR 0.4–3.8). The median frequency of follow-up appointments was 5 (IQR 2–11) in the retina service and 9 (4–18) across all subspecialty services; 15.9% (13 547) were discharged after their first appointment, while a small proportion required a high follow-up burden of 21–50 visits (7980, 9.3%) or >50 visits (1099, 1.3%) over the study period.

### Data Shifts

The RAMSEs dataset spans an 8.5-year period (2017–2025), during which temporal dynamics have caused some small distributional changes (“data shifts”).

**Figure 5:**
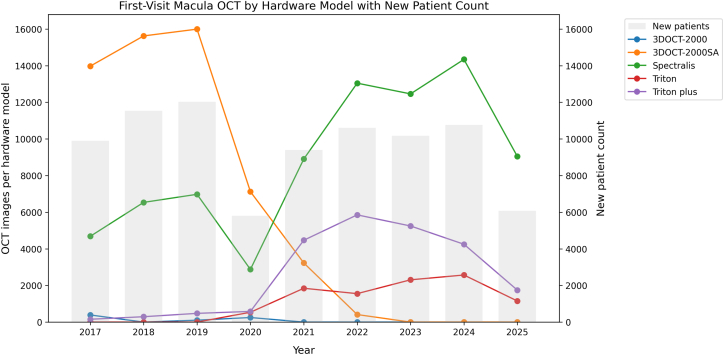
Line plots featuring total macula-centered OCT volumes at the baseline visit categorized by device model, with new patient counts displayed as a semitransparent bar chart for each year. The lines illustrate changes in image acquisition activity across device types, while the bars provide context on how many images represent a patient’s initial encounter in that year. Caveat: data from 2025 are incomplete (cutoff date in July 2025).

Moorfields Eye Hospital NHS Foundation Trust transitioned from earlier generation Topcon OCT devices to newer models with higher axial resolution and wider fields of view. Software upgrades also altered scan acquisition parameters, image file formats, and default segmentation algorithms. In addition, there was a shift in preference for OCT device manufacturers, with a sharp decline in Topcon OCT scans (especially 3DOCT-2000SA) and a corresponding rise in Spectralis OCT scans (Heidelberg) from 2020 onward (Fig 5).

The Covid-19 pandemic—which spanned March 2020 to July 2021 with 3 periods of national lockdowns—also precipitated severe health service disruptions, which reduced nonurgent clinic visits and accelerated the adoption of virtual clinics, potentially resulting in lower imaging throughput and potential shifts in disease severity at presentation. These system-level changes are also reflected in the RAMSEs cohort, with a sharp decline in new patient entries in 2020, and a slow recovery but incomplete recovery thereafter, with the numbers of new patients approaching but remaining below those observed prepandemic in 2019.

**Figure 6:**
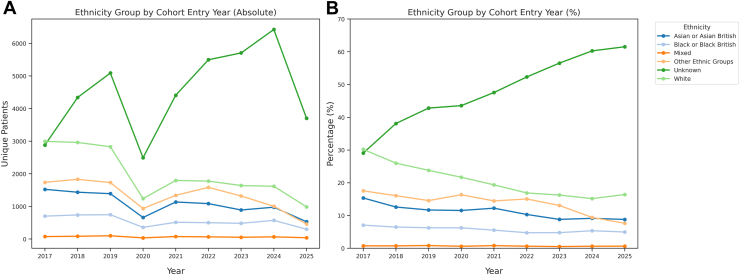
Ethnicity distribution by cohort entry year, presented as (**A**) absolute numbers and (**B**) percentages per year.

The population profile has stayed fairly constant in terms of age and sex, although there was an increasing trend toward patients declining to report ethnicity, contributing to the rising proportion of “unknown” ethnicity (Fig 6A, B).

## Strengths and Limitations

The RAMSEs dataset offers several key strengths. First, it leverages routinely collected clinical and imaging data from a sociodemographically diverse population, encompassing a broad spectrum of macular pathology and disease severity that mirrors that seen in real-world clinical practice in a secondary care setting. Access to both cross-sectional and longitudinal multimodal imaging at scale remains uncommon and is one of its key strengths for downstream research tasks. Second, MEH encompasses 27 networked sites, which enhances generalizability by capturing variability in referral patterns and clinician experience. Third, the availability of raw DICOM images and structured EHR data enable rich multimodal analyses.

Finally, dataset documentation was informed by the STANDING Together recommendations^14^ and the “Datasheets for Datasets” framework^15^ to ensure comprehensive documentation of provenance and processing, thereby promoting transparency and reproducibility. This includes the reporting of known or expected sources of bias, error, or other factors that may affect the generalizability or applicability of the dataset.

Here, we discuss these inherent limitations, their implications for dataset use, as well as attempts to mitigate them where possible.

### Known or Potential Exclusion Introduced by Data Collection

The RAMSEs cohort was derived from MEH’s network within Greater London, which is predominantly urban.^30^ In addition, while the ethnic diversity of this population is representative of the UK, the exact distribution in each category may differ in other UK regions. Patient demographics, referral pathways, and care protocols may also differ from nonurban or non-UK settings. In addition, this dataset reflects the range of patients referred to secondary eye care services and does not necessarily represent those seen and monitored in primary care.

RAMSEs excludes patients aged <18 years by design and is therefore not suitable for studying pediatric cohorts. Cohort entry criteria span the 2017–2025 period, which encompasses the coronavirus disease 2019 pandemic in 2020, during which national lockdowns and service disruptions may have influenced care-seeking behaviors and the availability of imaging and clinic capacity.

In addition, this dataset excludes all patients who exercised their right to opt out of having their data used for secondary purposes such as research. The national opt-out rate was 5.6% as of July 2025 (dataset cutoff date) and was approximately 7.0% within the London catchment area.^24^ The opt-out rates vary across demographic subgroups—this is higher in females compared to males (6.2% versus 4.9%) and lowest in young children (0–19 years) and oldest adults (>90 years).^24^ Because opt-outs preclude secondary use for research, the nonrandom missingness present here cannot be fully characterized or adjusted for. However, geographical variation in opt-out rates has been attributed to local socioeconomic and demographic factors, as well as contextual factors such as economic, political, and social environments.^31^

### Missing Data

With regards to relevant participant-level attributes, age was available for all patients in this cohort, and sex was absent for a miniscule proportion only (6, 0.007%). IMD scores were unavailable for 4991 patients (5.8%) due to invalid or suppressed postcodes, but the low proportion of missing data precluded meaningful subgroup analyses.

Ethnicity records were missing for 40 543 patients (47.4%), which was primarily due to nonrecording or nondisclosure at registration. Analysis of missingness patterns revealed no statistically significant differences in terms of sex (*P* = 0.323, chi-square test) or IMD decile (*P* = 0.259), although patients with missing ethnicity records were slightly younger (median 61, IQR 48–73 vs. 65, IQR 52–77, *P* < 0.001, Mann–Whitney *U* test). There was an increasing trend of missingness in ethnicity reporting over time. These findings suggest that ethnicity data are unlikely to be missing completely at random, and the potential for residual bias should be considered.

A substantial proportion of VA data was missing because only structured VA data fields were captured in this iteration of RAMSEs. Future versions will incorporate natural language processing-based extraction of VA measurements from free-text clinical letters.

No imputation has been applied to the dataset. Missing values were coded as “NA” (i.e. not applicable), and downstream analyses using this dataset can consider addressing them via appropriate strategies such as complete-case analysis, missing-indicator methods, or multiple imputation models as appropriate.

### Known or Potential Bias Caused or Exacerbated by Data Acquisition and Processing

The RAMSEs dataset prespecifies the inclusion of patients who attend the Medical Retina and Vitreoretinal Services who have a Topcon or Heidelberg macula-centered OCT scan taken at their initial visit. This means that patients who are managed clinically without an OCT scan are omitted, which may include those with severe positioning difficulties, mobility issues, or noncompliance with instructions. While this may induce an element of ascertainment bias from systematic differences in disease severity, comorbidities, or sociodemographic characteristics, the overall proportion is very small, particularly as imaging protocols for retinal services typically dictate OCT imaging, especially for those with suspected macular disease.

While the focus on 2 OCT device manufacturers may place some limits on generalizability, these are by far the most commonly used OCT devices in MEH. In addition, manufacturer selection is not site-dependent across MEH and is therefore not known to affect contextualized groups of interest. Future iterations of RAMSEs will incorporate OCT scans from additional vendors (e.g. Zeiss, Nidek) to enhance external validity and mitigate vendor-specific measurement biases.

### Known or Potential Bias in Derived/Assigned Data Labels

Diagnosis labels were initially derived from structured EHR records only, meaning that the level of completeness and consistency reflected some heterogeneity in clinicians’ coding practices. To mitigate missingness, these structured diagnosis labels will be augmented with data derived from natural language processing approaches applied to free-text clinic letters in future iterations of RAMSEs. In addition, data captured from routine clinical care may be prone to errors. Reference standard grading should therefore be performed on a representative subset of patient records and images to quantify label accuracy and assess for misclassification.

As ethnicity is self-reported, there is a potential for misclassification bias, but this is standard practice consistent across the NHS. Finally, socioeconomic status was assigned using IMD, which confers a risk of ecological fallacy as area-based indices may not accurately reflect individual circumstances, although this is an approach common to and widely accepted in health service research.

## Patient and Public Participation

Dataset creation was underpinned by robust patient and public involvement and engagement from the outset, in accordance with the STANDING Together recommendations.^14^ Prior to data curation, exploratory discussions with ophthalmology patients, carers, and members of the public were convened via focus groups, virtual forums, and one-to-one interviews to surface key themes around patient priorities and service improvements. Building on these insights, a dedicated patient advisory group representing diverse retinal conditions and health service experiences was formed to provide input on dataset curation, governance, and interpretation, and to provide feedback to ensure patient perspectives remain central to data analysis and downstream dissemination strategies. All outputs from RAMSEs, including summary reports, data visualizations, and plain language summaries, will be shared with patient advisory group members and via external communications.

## Dataset Access

RAMSEs may be available to external applicants via the INSIGHT Health Data Research Hub, an NHS-led ophthalmic bioresource designed to provide researchers with safe access to anonymized, routinely collected data with the aim of advancing research for patient benefit. Potential applicants can submit a data enquiry at the INSIGHT website (https://www.insight.hdrhub.org/insight-data). Provision of data is contingent on a data sharing agreement, adherence to strict governance requirements, appropriate funding on a cost recovery basis, and evaluation of a formal data use application by an independent Data Trust Advisory Board, which includes patients and public representatives and serves to ensure the request is appropriate and provides patient benefit to the NHS. Data are accessed within a dedicated platform for secure analysis of NHS data. Applicants must demonstrate adherence with information security frameworks and best practices in health data research. INSIGHT adheres to the FAIR principles (Findability, Accessibility, Interoperability, and Reusability) to promote reproducible and transparent research, and operates in full accordance with the UK Data Protection Act 2018 and UK Global Data Protection Regulation.

## Ethics and Governance

Curation of the dataset adhered to the tenets of the Declaration of Helsinki, and ethical approval was obtained from the relevant Institutional Review Board and ethics committee (West Scotland REC 4, 20/WS/0087). The requirement for explicit informed consent was waived as the dataset was fully anonymized and only retrospectively collected data were included, and patients who had previously opted out of having their confidential patient information used for research and planning have already been excluded in accordance with the NHS National Data Opt-out policy.^23^ The data collection process adhered to the tenets of the Declaration of Helsinki.

All data utilized in this study are presented in a research-ready format, where patient-level data were pseudoanonymized and anonymized through an irreversible deidentification process prior to access. Patient identifiers such as names, dates of birth, and addresses were removed, hospital or NHS numbers were hashed, and patient and clinical metadata underwent data treatment (e.g. date shifting, removal or bucketing of rare values) to prevent reidentification, in accordance with Data Privacy Laws and applicable guidance concerning anonymization issued by the UK Health Research Authority, the UK Information Commissioner’s Office, and standards recommended by the British Medical Journal.^32^ This is in line with INSIGHT’s governance processes and facilitates safe access to research data in keeping with the Office of National Statistics’ “Five Safes” framework.^33^

## Conclusion

We have developed a large multimodal real-world dataset comprising anonymized ophthalmic images and linked demographic and clinical metadata to study suspected macular disease in adult patients. RAMSEs was designed to address existing gaps in dataset size, distribution, and lack of critical metadata. This valuable resource can serve multiple purposes, including the development and robust clinical validation of AI models, obtaining insights into real-world patient pathways and outcomes, or to facilitate research relating to epidemiology or big data analytics.

## References

[1] Macular Society Macular conditions. https://www.macularsociety.org/macular-disease/macular-conditions/.

[2] Wong W.L., Su X., Li X. (2014). Global prevalence of age-related macular degeneration and disease burden projection for 2020 and 2040: a systematic review and meta-analysis. Lancet Glob Health.

[3] NHS Digital Hospital outpatient activity 2024-25. NHS England Digital. https://digital.nhs.uk/data-and-information/publications/statistical/hospital-outpatient-activity/2024-25.

[4] RCOphth The Way Forward: executive Summary. https://www.rcophth.ac.uk/wp-content/uploads/2021/12/RCOphth-The-Way-Forward-Executive-Summary-300117.pdf.

[5] Foot B., MacEwen C. (2017). Surveillance of sight loss due to delay in ophthalmic treatment or review: frequency, cause and outcome. Eye.

[6] RCOphth (2023). https://www.rcophth.ac.uk/wp-content/uploads/2023/03/2022-Ophthalmology-census-Facing-workforce-shortages-and-backlogs-in-the-aftermath-of-COVID-19.pdf.

[7] Arias L., Armadá F., Donate J. (2009). Delay in treating age-related macular degeneration in Spain is associated with progressive vision loss. Eye.

[8] Popescu M.L., Boisjoly H., Schmaltz H. (2011). Age-Related eye disease and mobility limitations in older adults. Invest Ophthalmol Vis Sci.

[9] Demmin D.L., Silverstein S.M. (2020). Visual impairment and mental health: unmet needs and treatment options. Clin Ophthalmol.

[10] Gupta P., Fenwick E.K., Man R.E.K. (2022). Different impact of early and late stages irreversible eye diseases on vision-specific quality of life domains. Sci Rep.

[11] Brown M.M., Brown G.C., Stein J. (2005). Age-related macular degeneration: economic burden and value-based medicine analysis. Can J Ophthalmol.

[12] Pezzullo L., Streatfeild J., Simkiss P., Shickle D. (2018). The economic impact of sight loss and blindness in the UK adult population. BMC Health Serv Res.

[13] Secinaro S., Calandra D., Secinaro A. (2021). The role of artificial intelligence in healthcare: a structured literature review. BMC Med Inform Decis Making.

[14] Alderman J.E., Palmer J., Laws E. (2024). Tackling algorithmic bias and promoting transparency in health datasets: the STANDING Together consensus recommendations. Lancet Digital Health.

[15] Gebru T., Morgenstern J., Vecchione B. (2021). Datasheets for datasets. arXiv. http://arxiv.org/abs/1803.09010.

[16] Khan S.M., Liu X., Nath S. (2021). A global review of publicly available datasets for ophthalmological imaging: barriers to access, usability, and generalisability. The Lancet Digital Health.

[17] Rozhyna A., Somfai G.M., Atzori M. (2024). Exploring publicly accessible optical coherence tomography datasets: a comprehensive overview. Diagnostics (Basel).

[18] Ong A.Y., Hogg H.D.J., Keane P.A. (2025). Cochrane corner: artificial intelligence for diagnosing exudative age-related macular degeneration. Eye (Lond).

[19] Ong A.Y., Taribagil P., Sevgi M. (2025). A scoping review of artificial intelligence as a medical device for ophthalmic image analysis in Europe, Australia and America. NPJ Digit Med.

[20] Hernandez-Boussard T., Bozkurt S., Ioannidis J.P.A., Shah N.H. (2020). MINIMAR (MINimum Information for Medical AI Reporting): developing reporting standards for artificial intelligence in health care. J Am Med Inform Assoc.

[21] De Fauw J., Ledsam J.R., Romera-Paredes B. (2018). Clinically applicable deep learning for diagnosis and referral in retinal disease. Nat Med.

[22] INSIGHT INSIGHT health data research hub for eye health. INSIGHT. https://www.insight.hdrhub.org.

[23] NHS Digital National data opt-out. NHS England Digital. https://digital.nhs.uk/services/national-data-opt-out.

[24] NHS England National Data Opt-Out open data dashboard. NHS England Digital. https://digital.nhs.uk/dashboards/national-data-opt-out-open-data.

[25] NHS NHS data model and dictionary. https://www.datadictionary.nhs.uk/data_elements/ethnic_category.html.

[26] NHS England Digital SNOMED CT. NHS England Digital. https://digital.nhs.uk/services/terminology-and-classifications/snomed-ct.

[27] Gov.uk English indices of deprivation 2019: mapping resources. GOV.UK. https://www.gov.uk/guidance/english-indices-of-deprivation-2019-mapping-resources.

[28] Day A.C., Donachie P.H.J., Sparrow J.M., Johnston R.L. (2015). The Royal College of Ophthalmologists' National Ophthalmology Database study of cataract surgery: report 1, visual outcomes and complications. Eye (Lond).

[29] Ong A.Y., Nguyen Q., Barai I. (2026). Developing a scalable pipeline for data extraction from clinical letters through resource-efficient prompt engineering. Res Square.

[30] Department for Environment, Food & Rural Affairs Official statistics: about the digest and rural definitions. GOV.UK. https://www.gov.uk/government/statistics/about-the-digest-and-rural-definitions/about-the-digest-and-rural-definitions.

[31] Tazare J., Henderson A.D., Morley J. (2024). NHS national data opt-outs: trends and potential consequences for health data research. BJGP Open.

[32] Hrynaszkiewicz I., Norton M.L., Vickers A.J., Altman D.G. (2010). Preparing raw clinical data for publication: guidance for journal editors, authors, and peer reviewers. BMJ.

[33] Denniston A.K., Kale A.U., Lee W.H. (2022). Building trust in real-world data: lessons from INSIGHT, the UK's health data research hub for eye health and oculomics. Curr Opin Ophthalmol.

